# Data on the structure, chemical state of carbon and discharge characteristics of multi-walled carbon nanotubes and composites based on them modified by pulsed ion beam

**DOI:** 10.1016/j.dib.2019.104108

**Published:** 2019-06-08

**Authors:** Petr M. Korusenko, Sergey N. Nesov, Sergey N. Povoroznyuk, Valery V. Bolotov, Yuri Sten'kin, Alexander I. Pushkarev, Ekaterina O. Fedorovskaya, Dmitry A. Smirnov, Konstantin E. Ivlev

**Affiliations:** a Omsk Scientific Center, Siberian Branch of the Russian Academy of Sciences, Karl Marx Avenue 15, 644040, Omsk, Russia; b Saint Petersburg State University, Universitetskaya Emb. 7/9, 199034, St. Petersburg, Russia; c Omsk State Technical University, Mira Avenue 11, 644050, Omsk, Russia; d Omsk State University, Mira Avenue 55a, 644077, Omsk, Russia; e National Research Tomsk Polytechnic University, Lenin Avenue 2a, 634028, Tomsk, Russia; f Novosibirsk State University, Pirogova Str. 2, 630090, Novosibirsk, Russia; g Nikolaev Institute of Inorganic Chemistry, Siberian Branch of Russian Academy of Sciences, Acad. Lavrentiev Ave. 3, 630090, Novosibirsk, Russia; h Institute of Solid State Physics, Dresden University of Technology, D-01069 Dresden, Germany

**Keywords:** Pulsed ion beam, Multiwalled carbon nanotubes, Core-shell, Composite, Li-ion batteries, XPS, XANES

## Abstract

The data presented in this article are related to the research article entitled “Structure and electrochemical characterization of SnO_x_/Sn@MWCNT composites formed by pulsed ion beam irradiation” (Korusenko et al., 2019). This article presents the effect of irradiation by pulsed ion beam (PIB) irradiation at various modes on the structure multi-walled carbon nanotubes (MWCNTs) and composites based on MWCNTs and tin oxide as well as cycling performance of these composites. The article also presents the results of the analysis of the structure of the electrodes, obtained on the basis of the initial and irradiated composites.

**Table:** Specifications table

Subject	Materials science, Physics, Chemistry
Specific subject area	Composite materials. Irradiation of materials by pulsed ion beam.
Type of data	Figures, Table
How data were acquired	XPS and XANES data (Russian-German beam line of BESSY II (Berlin) and experimental station PES-RGL). SEM data and quantitative elemental EDX analysis of composites (JEOL JSM 6610 LV scanning electron microscope). Charge–Discharge experiments were performed between 0.1 and 3.00 V versus Li/Li^+^ at a constant current density of 100 mA g^−1^ (galvanostatic mode) at room temperature. The A211-BTS-35-1U battery testing system was used to obtain measurements.
Data format	Raw, analyzed
Parameters for data collection	Irradiation of the MWCNTs and SnO_2-x_@MWCNT composites was carried out with a pulsed ion beam (PIB) at the TEMP-4M accelerator (15% H^+^, 85% C^+^, ion energy: 250 keV, pulse duration: 120 ns) under various impact parameters: energy density was 0.5 and 1 J/cm^2^; number of pulses was from 1 to 3.
Description of data collection	The changes in the structure and chemical state of MWCNTs and composites based on them by SEM, EDX and also XPS, XANES using synchrotron radiation were studied. XPS and XANES measurements were performed under ultrahigh vacuum conditions. SEM data were obtained using an accelerating voltage of 20 keV.
Data source location	Laboratory of physics of nanomaterials and heterostructures, Omsk Scientific Center, Siberian Branch of the Russian Academy of Sciences, Omsk, Russia
Data accessibility	With the article
Related research article	P.M. Korusenko, S.N. Nesov, V.V. Bolotov, S.N. Povoroznyuk, Yu.A. Sten'kin, A.I. Pushkarev, E.O. Fedorovskaya, D.A. Smirnov. Structure and electrochemical characterization of SnO_x_/Sn@MWCNT composites formed by pulsed ion beam irradiation. Journal of Alloys and Compounds Vol. 793, P. 723–731 (2019). https://doi.org/10.1016/j.jallcom.2019.04.066

**Table:** 

**Value of the data** • The results expand the base of experimental data on the impact of high-power pulsed ion irradiation on the structure of carbon materials •The data can be used to qualitatively assess changes of the structure and chemical state of carbon in the walls of carbon nanotubes under pulsed high-energy impacts. •The presented data on the structure and composition of composites modified by pulsed ion beam can be used in the development of methods for modifying porous nanostructured composite materials. •The results of the analysis of changes in the discharge capacity of the electrodes during the cycling process may be useful in developing new materials for the electrodes of lithium-ion batteries

## 1 Data

The dataset of this article provides information on the effect of pulsed ion beam irradiation at various parameters on the structure and chemical state carbon in MWCNTs and SnO_2_@MWCNTs composites. Also dataset provides information on structure and elemental composition as well as electrochemical performance of electrodes based on initial and irradiated composites.

**Fig. 1:**
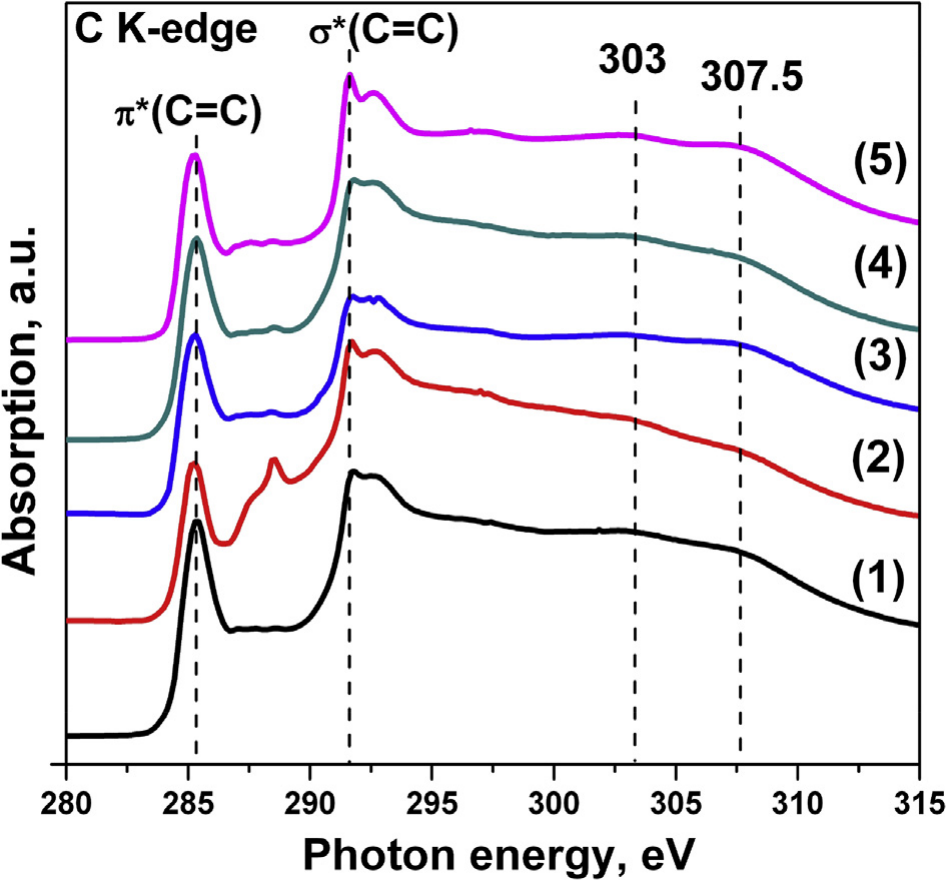
C K-edge XANES spectra of carbon for the MWCNTs before and after irradiation by pulsed ion beam with various energy density and number of pulses (n): (1) – initial MWCNTs; (2) – 0.5 J/cm^2^ (n = 1); (3) – 0.5 J/cm^2^ (n = 3); (4) – 1 J/cm^2^ (n = 1); (5) – 1 J/cm^2^ (n = 3).

Fig. 1 shows the XANES spectra of the initial and irradiated MWCNTs with different parameters. The intense maxima at the photon energies ∼285 and ∼291 eV, correspond to π*- and σ*- states of sp^2^-hybridized carbon in the walls of MWCNTs, respectively [2]. Carbon atoms bound to oxygen containing groups give features in the spectral region between π* and σ* resonances [2], [3]. As can be seen from Fig. 1, significant oxidation of carbon in the walls of MWCNTs is observed only for a sample once irradiated with a pulsed ion beam at an energy of 0.5 J/cm^2^ (curve 2).

**Fig. 2:**
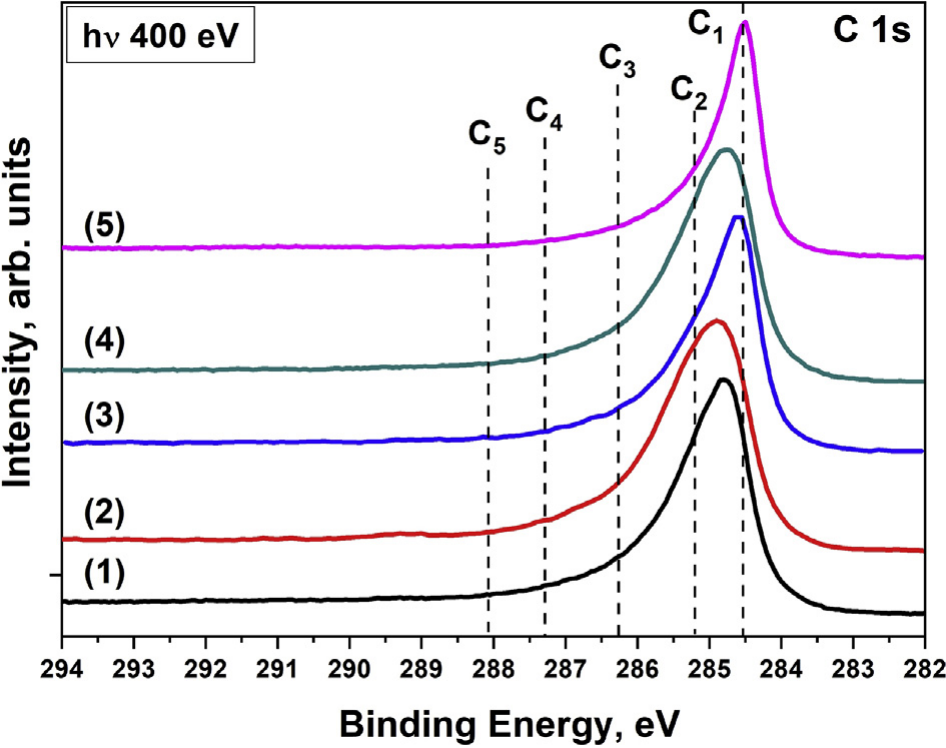
XPS C 1s spectra of the MWCNTs before and after irradiation by pulsed ion beam with various energy density and number of pulses (n): (1) – initial MWCNTs; (2) – 0.5 J/cm^2^ (n = 1); (3) – 0.5 J/cm^2^ (n = 3); (4) – 1 J/cm^2^ (n = 1); (5) – 1 J/cm^2^ (n = 3).

**Fig. 3:**
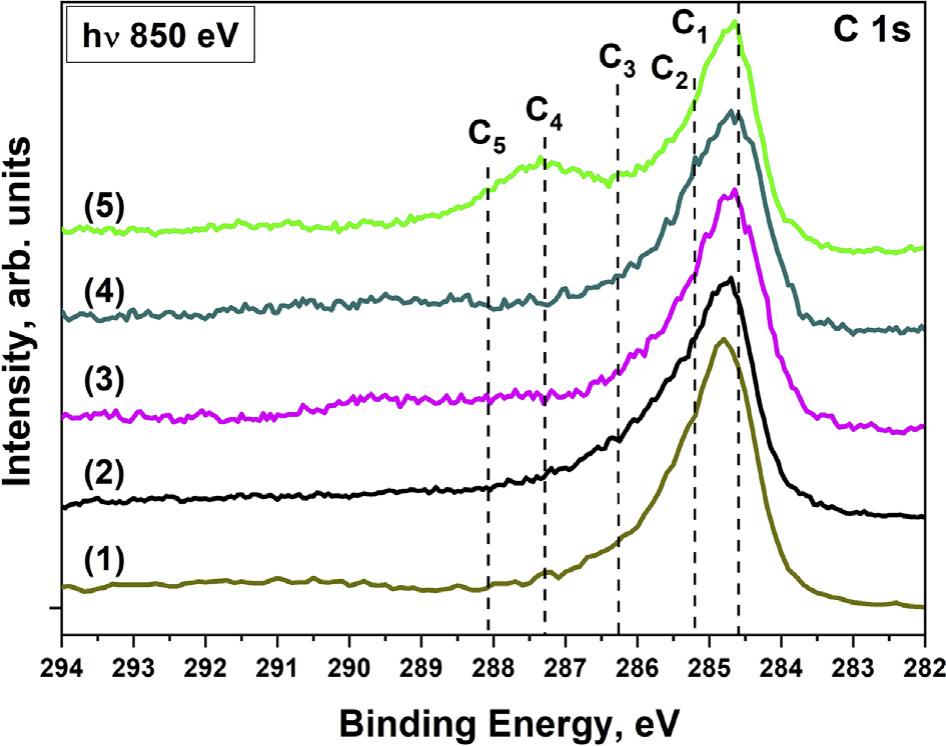
XPS C 1s spectra of the composites before and after irradiation by pulsed ion beam with various energy density and number of pulses (n): (1) – initial SnO_2-x_@MWCNTs; (2) – 0.5 J/cm^2^ (n = 1); (3) – 0.5 J/cm^2^ (n = 3); (4) – 1 J/cm^2^ (n = 1); (5) – 1 J/cm^2^ (n = 3).

Fig. 2, Fig. 3 show the carbon spectra for the MWCNTs and composites before and after irradiation by PIB with various modes, respectively. All spectra can be approximated by five components. The component C1 corresponds to sp^2^ carbon. The C2 corresponds to sp^3^ carbon and C–N bonds, as well as carbon being nearest to the oxygenated carbon (C*–C(O)) [4], [5]. The component C3 corresponds to carbon-oxygen species with single bonds (hydroxyl, epoxy and other groups). The components C4 and C5 correspond to carbon in C=O and COOH groups [5], [6]. It is seen that with an increase in the energy density and the number of irradiation pulses, an increase in the intensity of the C2–C5 components is observed. This indicates the oxidation of MWCNTs surface under the PIB impact. However, an increase in the number of pulses when exposed to a PIB leads to changes only when the composites are irradiated.

**Fig. 4:**
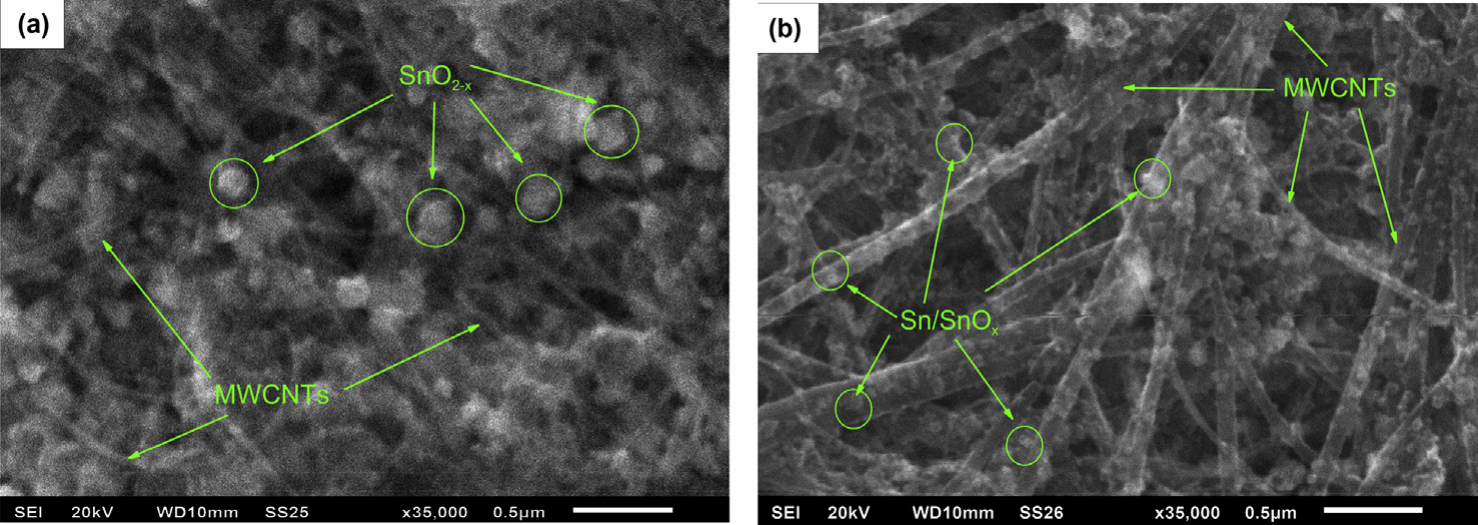
SEM images of the surface of the electrodes: (a) – initial SnO_2-x_@MWCNTs; (b) – composite after irradiation by pulsed ion beam at energy density of 0.5 J/cm^2^ (n = 3).

Fig. 4 shows the SEM images of the freshly prepared electrodes made from the initial composite and composite after irradiation with a pulsed ion beam. Also shown are EDX data carried out on various areas of the electrodes. As can be seen from Table 1, the amount of tin in the electrodes is almost the same.

**Table 1:** The EDX quantitative analysis for the initial and irradiated electrodes.

No. Point	Concentration, at.%					
	[C]	[O]	[F]	[P]	[Cu]	[Sn]
Initial SnO_2-x_@MWCNTs						
Point 1	73,41	7,31	14,94	0,32	0,63	3,39
Point 2	72,90	7,82	15,2	0,25	0,41	3,42
Point 3	66,39	7,82	18,5	0,39	1,29	5,61
Mean	80,56	5,34	8,42	0,53	0,72	4,41
0.5 J/cm^2^ (n = 3)						
Point 1	82,6	4,46	8,54	0,37	0,66	3,37
Point 2	79,92	8,51	8,33	0,49	0,44	2,31
Point 3	77,84	6,04	9,23	0,46	0,9	5,53
Point 4	81,88	2,35	7,61	0,8	0,9	6,46
Mean	70,9	7,65	16,21	0,32	0,77	4,14

**Fig. 5:**
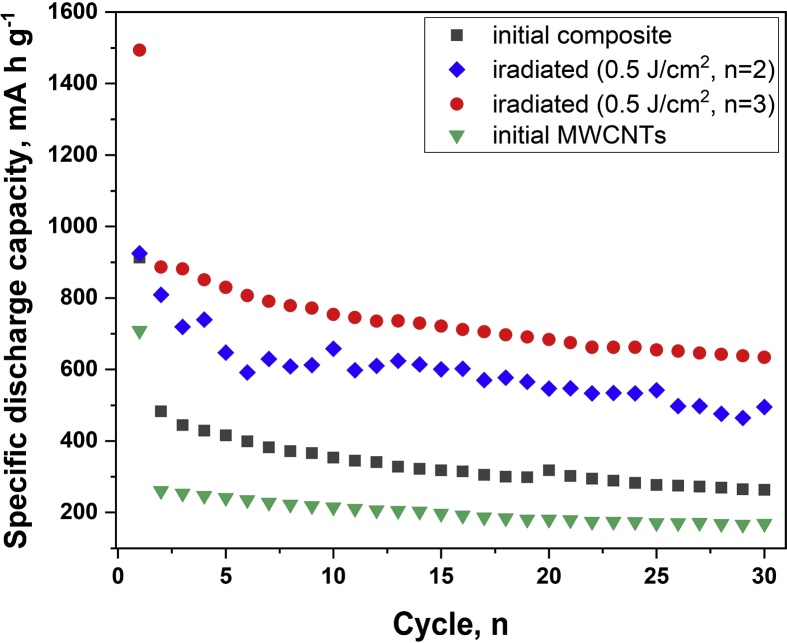
Capacities vs. cycle number for electrodes based on MWCNTs and the composites before and after irradiation by PIB.

Fig. 5 shows the discharge characteristics for electrodes formed on the basis of the initial and irradiated composites. As can be seen, the best characteristics are observed for the irradiated composite at energy density of 0.5 J/cm^2^ (n = 3) in which core-shell (Sn-SnO_x_) particles take place.

## 2 Experimental design, materials, and methods

The MWCNTs were synthesis through pyrolysis of a mixture of acetonitrile with ferrocene (100:1) performed in an argon flow (150 mL/min) at 800 °C for 30 min. Samples of composite SnO_2-x_@MWCNT were formed by thermal decomposition of a SnCl_2_·2H_2_O compound at a temperature of 550 °C, followed by vapor deposition on a heated substrate (MWCNTs/SiO_2_/Si) to 340 °C [7]. Pulsed ion beam TEMP-4M accelerator was used to modification of the MWCNTs and composites [1]. Irradiation was carrying out with various energy density and number of pulses (n): 0.5 J/cm^2^ (n = 1); 0.5 J/cm^2^ (n = 2); 0.5 J/cm^2^ (n = 3); 1 J/cm^2^ (n = 1); 1 J/cm^2^ (n = 3).

The working electrodes were formed by composite samples (85 wt%), carbon black (5 wt %) and PVDF (10 wt %) as the binder. This mixture was coated onto copper foil and was then annealed at 80 °C for 12 h under a vacuum. The cycling performances of the samples were measured using a CR2032 button cell. The counter electrode was made from metallic lithium. The electrolyte was a 1 M LiPF_6_ solution in mixture (EC:DMC = 1:1).

To study the local electronic structure of the MWCNTs and composites was carried out using XPS and XANES methods implemented at the Russian-German beam line at BESSY II (Berlin) and the PES-RGL experimental station. The XPS spectra were acquired at a photon energy of 400 eV, 850 eV and collected with the hemispherical analyzer PHOIBOS 150 using a pass energy of 15 eV. Step energy size was 0.05 eV. XPS spectra processing was performed using the CasaXPS software package. The absorption spectra were acquired by recording the leakage current from the sample. The monochromator resolutions for the carbon K-edge at hυ ∼285 eV was approximately ∼70 meV. The XANES spectra were normalized to the primary photon current from a gold covered grid recorded contemporaneously. The study of the electrode surfaces by the SEM method and quantitative EDX analysis was performed on the scanning electron microscope JEOL JSM 6610 LV. Charge–discharge experiments were performed between 0.1 and 3.00 V versus Li/Li^+^ at a constant current density of 100 mA g^−1^ in galvanostatic mode at RT.
